# Hemoglobin A1c and Arterial and Ventricular Stiffness in Older Adults

**DOI:** 10.1371/journal.pone.0047941

**Published:** 2012-10-30

**Authors:** Susan J. Zieman, Aruna Kamineni, Joachim H. Ix, Joshua Barzilay, Luc Djoussé, Jorge R. Kizer, Mary L. Biggs, Ian H. de Boer, Michel Chonchol, John S. Gottdiener, Elizabeth Selvin, Anne B. Newman, Lewis H. Kuller, David S. Siscovick, Kenneth J. Mukamal

**Affiliations:** 1 National Institute on Aging, National Institutes of Health, Bethesda, Maryland, United States of America; 2 Group Health Research Institute, Group Health, Seattle, Washington, United States of America; 3 Division of Nephrology-Hypertension, University of California San Diego, and Veterans Affairs San Diego Healthcare System, La Jolla, California, United States of America; 4 Division of Endocrinology, Kaiser-Permanente, Tucker, Georgia, United States of America; 5 Division of Aging, Brigham and Women's Hospital, Harvard Medical School, and Veterans Affairs Boston Healthcare System, Boston, Massachusetts, United States of America; 6 Departments of Medicine and Public Health, Weill Medical College of Cornell University, New York, New York, United States of America; 7 Departments of Biostatistics and Medicine, University of Washington, Seattle, Washington, United States of America; 8 Department of Medicine, University of Colorado Denver Health Sciences Center, Denver, Colorado, United States of America; 9 Division of Cardiology, University of Maryland School of Medicine, Baltimore, Maryland, United States of America; 10 Department of Epidemiology, Johns Hopkins Bloomberg School of Public Health, Baltimore, Maryland, United States of America; 11 Department of Epidemiology, University of Pittsburgh, Pittsburgh, Pennsylvania, United States of America; 12 Department of Medicine, Beth Israel Deaconess Medical Center, Boston, Massachusetts, United States of America; Omaha Veterans Affairs Medical Center, United States of America

## Abstract

**Objective:**

Arterial and ventricular stiffening are characteristics of diabetes and aging which confer significant morbidity and mortality; advanced glycation endproducts (AGE) are implicated in this stiffening pathophysiology. We examined the association between HbA_1c_, an AGE, with arterial and ventricular stiffness measures in older individuals without diabetes.

**Research Design & Methods:**

Baseline HbA_1c_ was measured in 830 participants free of diabetes defined by fasting glucose or medication use in the Cardiovascular Health Study, a population-based cohort study of adults aged ≥65 years. We performed cross-sectional analyses using baseline exam data including echocardiography, ankle and brachial blood pressure measurement, and carotid ultrasonography. We examined the adjusted associations between HbA_1c_ and multiple arterial and ventricular stiffness measures by linear regression models and compared these results to the association of fasting glucose (FG) with like measures.

**Results:**

HbA_1c_ was correlated with fasting and 2-hour postload glucose levels (r = 0.21; p<0.001 for both) and positively associated with greater body-mass index and black race. In adjusted models, HbA_1c_ was not associated with any measure of arterial or ventricular stiffness, including pulse pressure (PP), carotid intima-media thickness, ankle-brachial index, end-arterial elastance, or left ventricular mass (LVM). FG levels were positively associated with systolic, diastolic and PP and LVM.

**Conclusions:**

In this sample of older adults without diabetes, HbA_1c_ was not associated with arterial or ventricular stiffness measures, whereas FG levels were. The role of AGE in arterial and ventricular stiffness in older adults may be better assessed using alternate AGE markers.

## Introduction

Increased arterial and ventricular stiffness are hallmarks of aging and its attendant comorbidity [1]. Clinical measures of arterial stiffness, such as elevated pulse pressure (PP) and isolated systolic hypertension (ISH), are associated with greater risk for cardiovascular disease (CVD), chronic kidney disease, dementia, and mortality. Ventricular stiffness is associated with left ventricular hypertrophy (LVH) and delayed early diastolic filling which, in turn, increase the risk of heart failure (HF), and death [2]. People with diabetes, both young and old, carry an even higher risk of arterial and ventricular stiffness and its cardiovascular sequelae [3], [4].

Age-associated arterial changes include increased intima-medial thickness (IMT), smooth muscle hypertrophy, collagen accrual and cross-linking, fibrosis, and inflammation [1]. These aging changes are, in part, due to time-related exposure to and accrual of advanced glycation endproducts (AGE), irreversibly glycated proteins, which, in turn, stimulate systemic inflammation, oxidative stress, fibrosis, extracellular matrix remodeling, tissue injury modulation and lipid deposition in the arterial wall [5]. Elevated blood glucose concentrations favor AGE formation. Thus, AGE may play an important role in arterial and ventricular stiffness associated with aging even in individuals without diabetes, but with evidence of elevated blood glucose levels.

Glycosylated hemoglobin (HbA_1c_) is an AGE that serves clinically as a marker of average glycemia in individuals with diabetes and recently has been added as a diagnostic criterion for diabetes [6]. This is based on several large cohort studies demonstrating the positive association of HbA_1c_ levels with incident cardiovascular events, stroke and mortality in those with and, in some cases, those without diabetes [7], [8], [9], [10]. HbA_1c_ levels are also positively correlated with subclinical arterial disease (carotid intimal medial thickness [cIMT] and ankle-brachial index) in people with diabetes [11], [12], but the MESA cohort found only a weak correlation between HbA1c and cIMT and coronary calcium scores in subjects without diabetes (mean age 63±10 yrs) [13]. As an AGE of hemoglobin in red blood cells (half-life of approximately 100 days), HbA_1c_ may not reflect ambient glycemia over longer time periods. Nevertheless, it represents a potential useful biomarker with which to evaluate whether AGE are associated with measures of arterial and ventricular stiffness. To our knowledge, no previous large-scale study has comprehensively evaluated the association of the full range of values of this AGE with multiple non-invasive measures of arterial and ventricular stiffness, especially in a population-based study of older adults.

To address these issues, we examined the cross-sectional associations of HbA_1c_ with several markers of ventricular and arterial stiffness and atherosclerosis among older adults free of prevalent diabetes in the Cardiovascular Health Study (CHS).

## Methods

### Study Population

CHS is a NHLBI-sponsored, population-based, longitudinal study of clinical and subclinical CVD and its risk factors among 5888 community-dwelling older adults in the U.S. [14], [15] Utilizing Medicare eligibility lists, 5,201 ambulatory, non-institutionalized men and women were contacted and enrolled from four U.S. communities (Allegheny County, Pennsylvania; Forsyth County, North Carolina; Sacramento County, California; and Washington County, Maryland) in 1989–90. The Wake Forest University Health Sciences Institutional Review Board (IRB), University of California Davis IRB, Johns Hopkins Bloomberg School of Public Health IRB, and University of Pittsburgh IRB approved the study, and all participants provided written informed consent. Participants underwent a standardized clinic examination, laboratory evaluation, echocardiography, and questionnaires on health status, medical history, and cardiovascular risk factors [16], [17].

HbA_1c_ was measured in a substudy of 1,094 participants of the 1,305 recruited from Forsyth County at the baseline examination in 1989–90. Of these, we excluded 62 participants with chronic atrial fibrillation, 39 with moderate or severe mitral regurgitation, 4 with moderate or severe aortic stenosis, and 6 with aortic insufficiency, in whom echocardiographic measures were considered less reliable measures of arterial or ventricular stiffness. To minimize the influence of outliers and potentially errant values, we also excluded 14 participants with values above an arbitrary HbA_1c_ threshold of 14%. Lastly, we excluded 8 participants missing information on diabetes status.

Because our primary interest was in the association of HbA_1c_ with vascular stiffness among non-diabetic individuals, we excluded 131 persons with prevalent diabetes on the basis of the previous definition of diabetes as fasting glucose≥126 mg/dL or use of insulin or oral hypoglycemic agents, providing an analytic sample of 830. Participants with prevalent diabetes by this definition were included as a separate category in secondary analyses in which HbA_1c_ was categorized in approximate tertiles. In those analyses, the threshold for the highest tertile was 6.5%, so that the highest category corresponded to the current diagnostic threshold for diabetes, creating categories of low and intermediate HbA_1c_, high HbA_1c_ but without other criteria for diabetes, and diabetes by previous criteria.

### Measurement of HbA_1c_

HbA_1c_ was measured in previously frozen whole blood samples from 1989–90 stored at −70°C. A standard kit affinity column method was employed (normal values ranged from 2.9–5.1%) and standardized against high pressure liquid chromatography procedures. The Wake Forest University Clinical Pathology Department performed these assays with regular monitoring for quality assurance.

### Measurement of Arterial and Ventricular Stiffness and Atherosclerosis

We tested the relationship between HbA_1c_ and several non-invasive peripheral measures of arterial stiffness, including PP and SBP. Additional estimates of total arterial stiffness, (effective arterial elastance (Ea), PP/Stroke Volume) were calculated utilizing echocardiographic data described later. Finally, we included two measures of subclinical vascular disease – carotid IMT and ankle-brachial index (ABI), which we considered to reflect both arterial stiffness and atherosclerosis.

Study technicians measured resting brachial blood pressure according to a standardized protocol [18], and we used the average of the first and second readings for systolic and diastolic values. Pulse pressure was calculated as the difference between systolic and diastolic values. Vascular ultrasound of the internal and common carotid arteries was used to determine IMT [19]. We determined carotid IMT as the mean of the maximum wall thickness for near and far walls on both the left and right carotid arteries. We used both internal and common carotid IMT in our analyses. We calculated ABI as the ratio of the lower of ankle pressures taken in the right and left legs divided by the average of two right arm blood pressure measurements [20]. Individuals with ABI>1.40 were excluded from this analysis (n = 11).

Participants underwent routine M-mode, 2-dimensional, and Doppler echocardiographic examinations at baseline using the Toshiba SSH-160A (Toshiba American Medical Systems, Tustin, CA) equipped with 2.5 MHz and 3.75 MHz transducers [17]. American Society of Echocardiography recommendations were used to guide two-dimensionally directed M-mode measurements of LV mass [21]. Mitral flow velocity including early and late (atrial) peak filling and regurgitation was assessed by Doppler.(21) Stroke volume (SV) was estimated using the left ventricular velocity-time integral and calculated outflow tract area as previously described [22]. Trained readers interpreted stored echocardiographic images centrally at the CHS Echo Reading Center. Quality control measures included standardized training of sonographers and readers, observation of technicians by a trained echocardiographer, periodic blind duplicate readings, phantom studies, and quality control adults.

Based upon echocardiographic measurements, we calculated several variables related to arterial and ventricular stiffness. Effective arterial elastance (Ea), an estimate of total arterial compliance was calculated using estimated end-systolic pressure (ESP) as ([2*SBP]-DBP)/3 and stroke volume index (SVI) as SV/body surface area (BSA); Ea was calculated as the ratio of ESP to SVI. Total arterial load (PP/SV) was calculated from brachial PP and echocardiographic SV. Ventricular structure was represented by left ventricular (LV) mass and ventricular stiffness by the ratio of early to atrial filling (E/A ratio) on mitral pulsed Doppler inflow tracings and end-systolic elastance (Ees) was calculated as the ratio of ESP to LV end-systolic volume (by echocardiogram)/BSA.

### Other Measurements

Body mass index (BMI) was calculated from technician-measured height and weight. Participants were classified as smokers if they reported smoking within the previous 30 days or identified themselves as smokers. Former and never smokers were classified based on self-reported smoking status and reported no cigarette smoking in the past 30 days. Blood collection, processing, and laboratory methods for fasting glucose, lipid measurements, C-reactive protein, and creatinine are described elsewhere [16], [23]. Participants also underwent standard oral glucose tolerance testing (OGTT) at baseline. Study staff performed detailed medication inventories to obtain information on medication use [24]. We defined hypertension as SBP≥140 mmHg, DBP≥90 mmHg, or the use of hypertension medications.

### Statistical Analysis

In descriptive analyses, we categorized HbA_1c_ into approximate tertiles and evaluated differences across categories using analysis of variance for continuous variables or χ^2^ tests for categorical variables. We used linear regression models to examine the association between HbA_1c_ as continuous variable and measures of arterial and ventricular stiffness, initially adjusting for age, gender, and race. For comparison, we then repeated these models substituting fasting blood glucose for HbA_1c_. The various outcome measures were standardized (i.e., expressed in 1-standard deviation increments, which were the equivalent of 1% in HbA_1c_ and 10 mg/dl in fasting blood glucose) for comparability across measures. In multivariable models, we further adjusted for BMI, smoking status, use of antihypertensive medication, use of lipid-lowering medication, and levels of total cholesterol, high-density lipoprotein cholesterol, C-reactive protein, and creatinine. The model for LV mass was also adjusted for BSA.

We next examined the association between categories of HbA_1c_ and dichotomized measures of subclinical disease using logistic regression. We adjusted for the same covariates as in linear regression models and used previously established cutpoints for IMT (the 80^th^ percentile), ABI (0.9), and blood pressure (systolic>140; diastolic>90 mm Hg) to categorize subclinical disease. We also examined these same measures of subclinical disease in those with prevalent diabetes defined by glucose or medication use (n = 131) for comparative purposes.

We also determined total glycated hemoglobin, calculated as the product of HbA_1c_ multiplied by total hemoglobin [25], but this was highly correlated with HbA_1c_ itself (ρ 0.85) and did not yield materially different associations than HbA_1c_ with other glycemic measures. Based upon the possibility of sex-specific associations of HbA_1c_ with subclinical disease, we examined interactions of sex and HbA_1c_ on measures of vascular and ventricular stiffness, but none of these were significant (p>0.077).

Analyses were performed using STATA 10.1 (College Station, Texas). A.K. and M.L.B. had full access to all data and assumes responsibility for data analyses.

## Results

Table 1 displays the characteristics of eligible participants according to categories of HbA_1c_. Compared to the lowest category, those with higher HbA_1c_ were more often black, had greater BMI, and had higher CRP concentrations. Participants with higher HbA_1c_ also had greater LV mass, while other vascular measurements were similar across HbA_1c_ categories in bivariate analysis. HbA_1c_ was moderately correlated with both fasting and 2-hour glucose levels (Pearson ρ 0.21; p<0.001 for both).

**Table 1:** Characteristics of Wake Forest CHS Participants without Diabetes Defined by Glucose or Medication, According to Categories of HbA_1c_.

	Category of HbA_1c_			
	3.7–5.6%	5.7–6.4%	6.5–12.8%	P value
Median HBA1_c_	5.3	6.0	6.9	
	(n = 278)	(n = 293)	(n = 259)	
**Demographics**				
Age (yrs)	71.8 (5.3)	71.7 (5.1)	72.3 (5.4)	0.22
Male	118 (42.5%)	117 (36.6%)	89 (38.4%)	0.33
Black	11 (4.0%)	19 (5.9%)	30 (12.9%)	<0.001
Current smoker	35 (12.6%)	39 (12.2%)	47 (20.3%)	0.02
**Risk Factors**				
Body-mass index (kg/m^2^)	25.0 (4.6)	25.3 (3.9)	26.5 (4.3)	<0.001
Total cholesterol (mg/dl)	204.8 (35.6)	209.9 (36.6)	211.4 (38.2)	0.09
HDL cholesterol (mg/dl)	55.4 (16.4)	54.2 (16.3)	52.2 (13.1)	0.07
Systolic blood pressure (mmHg)	135.0 (21.6)	133.3 (21.2)	137.4 (19.7)	0.08
Diastolic blood pressure (mmHg)	80.0 (10.4)	71.2 (10.5)	73.1 (10.1)	0.09
Fasting glucose (mg/dL)	96.7 (9.4)	97.9 (9.1)	102.2 (9.7)	<0.001
2-hour glucose (mg/dL)	124.5 (34.5)	132.2 (44.2)	152.6 (48.3)	<0.001
Creatinine (mg/dl)	1.03 (0.25)	1.03 (0.25)	1.05 (0.35)	0.56
**Vascular Measures**				
Pulse pressure (mmHg)	63.0 (18.2)	62.1 (17.4)	64.2 (16.9)	0.36
Common carotid IMT (mm)	1.03 (0.20)	1.03 (0.22)	1.05 (0.20)	0.68
Internal carotid IMT (mm)	1.33 (0.51)	1.33 (0.55)	1.43 (0.55)	0.06
Ankle brachial index	1.08 (0.15)	1.09 (0.14)	1.08 (0.14)	0.84
ABI<.9 (vs. ≥.9–1.4)	26 (10.0%)	25 (8.0%)	18 (7.9%)	0.63
**Ventricular Measures**				
Left ventricular mass (gm)	144.6 (28.1)	143.0 (28.6)	149.9 (30.0)	0.02
E/A ratio	0.92 (0.28)	0.95 (0.32)	1.26 (5.4)	0.34
**Mixed Measures**				
Pulse pressure/stroke volume	0.83 (0.31)	0.82 (0.28)	0.85 (0.30)	0.64
Effective-Arterial Elastance (esp/svi)	1.50 (0.46)	1.47 (0.40)	1.54 (0.42)	0.28
Ratio of End-Arterial to End-Systolic Elastance	0.25 (0.09)	0.24 (0.09)	0.24 (0.10)	0.55
**Prevalent Diagnoses**				
Hypertension	158 (56.8%)	173 (54.1%)	147 (63.4%)	0.09
Stroke	13 (4.7%)	5 (1.6%)	5 (2.2%)	0.06
Coronary Heart Disease	55 (19.8%)	41 (12.8%)	38 (16.4%)	0.07
Chronic Kidney Disease (eGFR<60)	25 (9.9%)	37 (13.0%)	41 (20.0%)	0.007
Congestive Heart Failure	5 (1.8%)	6 (1.9%)	3 (1.3%)	0.86

Data are means (± standard deviations) or proportions.

Table 2 shows the associations of HbA_1c_ with multiple measures of vascular and ventricular stiffness. In both unadjusted and adjusted models, we did not observe any significant associations of measures of vascular stiffness and HbA_1c_. There was an association of borderline statistical significance with internal carotid IMT in age-, gender-, and race-adjusted models; however, this appeared to be mainly confounded by the positive associations of HbA_1c_ with BMI, smoking status, and C-reactive protein and its inverse association with high-density lipoprotein cholesterol, so was attenuated and no longer statistically significant in the final adjusted model. Sensitivity analyses additionally adjusted for weight loss/previous BMI or excluding participants on hypoglycemic medications did not render any significant relationships. In contrast to HbA_1c_, fasting blood glucose was significantly positively associated with SBP, DBP, PP and LV mass in the multivariate model.

**Table 2:** Associations of HbA1c and Fasting Glucose with Measures of Arterial and Ventricular Stiffness among Individuals free of Diabetes by Fasting Glucose or Medication Use.

			Standardized Increment per 1% Increase in HbA_1c_						Per 10 mg/dl Fasting Glucose	
			Crude model		Age-gender-race- adjusted model		Multivariable model^1^		Multivariable model	
Vascular Measures	Standard Deviation	No. of subjects	β	p-value	β	p-value	β	p-value	β	p-value
Systolic blood pressure	21 mmHg	829	0.019	0.59	0.005	0.89	−0.012	0.73	0.13	0.001
Diastolic blood pressure	10.4 mmHg	829	−0.0003	0.99	−0.005	0.89	−0.02	0.58	0.11	0.006
Pulse pressure	17.6 mmHg	829	0.023	0.52	0.009	0.80	−0.003	0.93	0.10	0.01
										
Common carotid IMT	0.21 mm	828	0.043	0.22	0.038	0.26	0.004	0.91	0.0002	0.99
Internal carotid IMT	0.54 mm	827	0.073	0.04	0.075	0.03	0.031	0.36	0.01	0.73
Ankle brachial index	0.14	803	−0.022	0.56	0.018	0.60	0.047	0.17	0.01	0.70
Left ventricular mass^2^	29 gm	813	0.024	0.28	0.025	0.24	0.017	0.40	0.05	0.02
Effective arterial elastance (Ea)	0.43	500	0.032	0.49	0.006	0.89	−0.003	0.95	0.02	0.65
Pulse pressure/stroke volume	0.30	501	0.008	0.85	−0.023	0.61	−0.010	0.83	−0.004	0.93
E/A ratio	2.9	813	0.058	0.58	0.015	0.88	0.043	0.69	0.14	0.24
End-systolic elastance (Ees)	1.9	500	0.068	0.45	−0.013	0.89	−0.026	0.77	0.09	0.33
Ea/Ees	0.09	500	−0.005	0.26	−0.002	0.58	−0.001	0.85	0.0004	0.93

1 Adjusted for age, gender, race (black, other), BMI, smoking status (never, former, current), total cholesterol, HDL-cholesterol, antihypertensive medications, lipid-lowering medications, C-reactive protein, and creatinine.

2 LV mass further adjusted for body surface area.

Table 3 shows the association (odds ratios) of categories of HbA_1c_ with carotid IMT, ABI, and hypertension, as well as the category of prevalent diabetes defined by fasting glucose or medication use. HbA_1c_ was not associated with any of these in directions consistent with greater stiffness, and paradoxically, it was associated with a lower prevalence of low ABI, although it was not associated with ABI in linear analyses shown in Table 2. In contrast, those with prevalent diabetes defined by glucose or medication had a substantially higher prevalence of hypertension in the multivariable model compared to the reference group. The prevalence of common carotid IMT ≥80% was also modestly higher among participants with diabetes, but this increase was not statistically significant in the fully adjusted model. No significant increases in prevalence were seen for internal carotid IMT ≥80% or ABI<0.9 among those with prevalent diabetes defined by medication use or fasting glucose.

**Table 3:** Odds ratios and 95% confidence intervals for selected measures of stiffness/atherosclerosis, according to categories of HbA1c and prevalent diabetes.

	No. of subjects	HbA_1c_ 3.7–5.6%	HbA_1c_ 5.7–6.4%	HbA_1c_ 6.5–12.8%	Prevalent diabetes defined by glucose or medication use (n = 131)
Common carotid IMT ≥80%					
age-gender-race- adjusted model	957	1.0	0.89 (0.57–1.37)	1.04 (0.66–1.65)	1.78 (1.08–2.92)
multivariable model^1^	951	1.0	0.86 (0.55–1.34)	0.84 (0.52–1.35)	1.55 (0.91–2.63)
Internal carotid IMT ≥80%					
age-gender-race- adjusted model	954	1.0	1.14 (0.74–1.77)	1.41 (0.89–2.22)	1.37 (0.81–2.33)
multivariable model^2^	948	1.0	1.10 (0.70–1.73)	1.15 (0.71–1.87)	1.04 (0.59–1.84)
ABI <.9 (vs. 0.9–1.4)					
age-gender-race- adjusted model	926	1.0	0.81 (0.44–1.46)	0.64 (0.33–1.24)	1.27 (0.63–2.54)
multivariable model^2^	921	1.0	0.79 (0.42–1.48)	0.45 (0.22–0.93)	0.95 (0.44–2.03)
Hypertension					
age-gender-race- adjusted model	961	1.0	0.90 (0.65–1.25)	1.28 (0.89–1.84)	2.65 (1.64–4.30)
multivariable model^2^	955	1.0	0.85 (0.61–1.19)	1.05 (0.71–1.54)	2.04 (1.23–3.38)

1 Adjusted for age, gender, race (black, other), BMI, smoking status (never, former, current), total cholesterol, HDL cholesterol, antihypertensive medications, lipid-lowering medications, C-reactive protein, and creatinine.

2 Adjusted for all covariates in other multivariable models except for antihypertensive medications.

## Discussion

In this cross-sectional study of community-living, older adults, blood values of HbA_1c_ were not associated with measures of arterial or ventricular stiffness. In contrast, systolic, diastolic, and pulse pressures and LV mass were positively associated with fasting blood glucose. Thus, it would appear that as a relatively short-lived AGE, HbA_1c_ may not serve well as a biomarker for early subclinical atherosclerotic or hypertensive changes in older non-adults without diabetes.

This finding contrasts with the positive association of HbA_1c_ and carotid IMT and ABI in subjects with known diabetes of the ARIC cohort, reflecting the possible importance of the duration of hyperglycemia exposure [11], [12]. Consistent with this hypothesis, only a weakly positive association between HbA_1c_ and common and internal cIMT was observed in those without known diabetes in MESA. Although this relationship was not seen in our cohort, it is important to note that CHS subjects were significantly older and this measure of HbA_1c_ was performed late in life. This may also explain the lack of association of HbA_1c_ with incident clinical cardiovascular events in CHS [26].

An important consideration in the evaluation of HbA_1c_ as an early biomarker for subclinical cardiovascular disease is its relatively rapid turnover of months rather than years due to the limited half-life of erythrocytes. This stands in contrast to the glycation of structural proteins such a collagen, a major component of vascular and ventricular structures, which renders them resistant to hydrolysis resulting in an overabundance of crosslinked proteins. As a result, single levels of HbA1c, even if well measured, may not necessarily reflect long-term AGE accumulation. Moreover, how closely correlated serum HbA1c and structural glycated proteins are remains unclear. Future studies using circulating AGEs with longer half-lives, such as carboxymethyl and carboxyethyl lysine, may shed light on this topic [27].

The strongly positive associations that we report between fasting blood glucose and systolic, diastolic, and pulse pressures support the theory that elevated blood glucose, possibly through AGEs, impact arterial stiffness, a prominent feature of diabetes and aging. The positive association between fasting blood glucose and LV mass are also consistent with findings in animal models in which induced diabetes leads to increased ventricular pressure, myocyte hypertrophy, and collagen accrual, all mediated by AGEs [28].

Our findings point to the need for reproducible and inexpensive measures of blood AGEs that can be applied to epidemiological settings to evaluate their potential as early biomarkers of subclinical atherosclerotic disease and early arterial and ventricular stiffening, not only in persons with diabetes, but also among older individuals in general population. The importance of identifying a long-term AGE measure is twofold: (1) to elucidate the pathophysiology which renders arterial and ventricular stiffness a prominent cause of morbidity and mortality in older adults, and (2) in those with diabetes and to identify targets for therapy to reduce stiffness. Novel therapeutics that reverse AGE crosslinks have been tested but are not currently available [29]. Given the rapidly expanding population of older adults and prevalence of diabetes, and the burden of cardiovascular disease in these populations, the relationship of long-term AGE with vascular stiffness should be a high priority for future research.

An implication of our findings relates to the adoption of HbA_1c_ as a diagnostic tool for diabetes. The choice of 6.5% as the threshold for diagnosis results in great part from the fairly linear cross-sectional association of HbA_1c_ with prevalence of retinopathy that begins at that level [6]. The third category of HbA_1c_ in our analyses (approximately the upper tertile of HbA_1c_) corresponds to the subset of individuals who would be classified as having diabetes only by the HbA_1c_ criterion. This criterion appears useful even in those without known diabetes in predicting future risk of diabetes, coronary heart disease, stroke and death in other cohorts [10]. However, our findings of a similar degree of vascular stiffness across the three HbA_1c_ tertiles in CHS suggests caution in using HbA_1c_ as a surrogate marker for subclinical atherosclerotic and microvascular disease in older adults without known diabetes.

One of the strengths of CHS is the large and diverse group of subclinical vascular phenotypes available. Despite the availability of several measures, we found no discernible association between HbA_1c_ and any outcome, whether related to blood pressure, ventricular mass, ventricular stiffness, or vascular stiffness, but were able to corroborate the elevated prevalence of hypertension in CHS participants with prevalent diabetes compared to those without diabetes in the lowest HbA_1c_ category.

Our study has limitations. We relied on single measures of both HbA_1c_ and outcomes in this cross-sectional analysis. Blood pressure was also measured in duplicate, but triplicate (or greater) replications are now common in observational studies. Inherent variability in these measures within and between individuals may have attenuated their relationship with outcome. Results should be evaluated within the context of the 95% confidence intervals, and we do not have information on fasting glucose or HbA_1c_ prior to baseline. In addition, HbA_1c_ was only moderately correlated with fasting and post-load glucose measurements in this population without diabetes; previous studies vary widely on the correspondence of HbA_1c_ with fasting glucose but the moderate correlation observed here has been reported previously. It is possible that studies with repeated measures of HbA_1c_ may reveal modest associations that this study missed. Additionally, echocardiographic measures of left ventricular diastolic function increasingly rely upon tissue Doppler, which was not available during the studies in this analysis, nor were alternative measures of arterial stiffness, such as pulse wave velocity or the ß stiffness index. Nevertheless, the non-invasive measures of ventricular and vascular stiffness used herein have shown validity in other population-based cohorts [30], [31].

Although CHS is a population-based cohort study, participants were generally healthy, community-dwelling older adults. Moreover, this substudy was conducted at the Wake Forest field center and does not include participants from the other three centers. As a result, our findings should be generalized to other populations with an appropriate degree of caution.

In summary, our data do not provide evidence for an association between HbA_1c_ and arterial and ventricular stiffness or subclinical atherosclerosis in a sample of community-living older adults. It is possible that the half-life of HbA_1c_ is too short, and that measurement of more stable longer lasting AGEs may be required to detect associations with arterial stiffness and associated comorbidities in individuals without diabetes. Alternatively, factors other than AGE may be predominant determinants of arterial and ventricular stiffness in this population, or circulating AGE may not reflect AGE deposition in structural proteins.
